# Chylous ascites: Case report of a rare presentation of blunt abdominal trauma

**DOI:** 10.1016/j.ijscr.2020.11.134

**Published:** 2020-11-30

**Authors:** Vânia Fernandes, Jacinta Queirós, Carlos Soares

**Affiliations:** General Surgery Department, Centro Hospitalar Tâmega e Sousa, Penafiel, Portugal

**Keywords:** Case report, Chylous ascites, Blunt abdominal trauma, Triglycerides, Low-fat diet

## Abstract

**Introduction:**

Chylous ascites is the accumulation of a milk-like peritoneal fluid rich in triglycerides, due to the presence of intestinal lymph in the abdominal cavity. The most common causes of chylous acites in adults are abdominal malignancy and cirrhosis. Very few cases of chylous ascites associated to blunt abdominal trauma have been published in the literature.

**Clinical case:**

A 27-year-old, female patient was admitted to the emergency department (ED) with abdominal pain due to a deceleration-type traffic accident. During surveillance the patient presented a progressive decrease in hemoglobin levels and an increase in free intra-abdominal fluid detected on computed tomography scan. The patient underwent an exploratory laparoscopy and a milky-looking peritoneal fluid was identified. The diagnosis of chylous ascites was confirmed by the determination of increased triglyceride levels in the peritoneal fluid. A low-fat diet, with a restriction of long-chain triglycerides, was started in the post-operative period and the patient presented a progressive decrease in abdominal drainage. The patient had a favorable clinical and analytical evolution and was discharged on the fifth post-operative day.

**Discussion:**

Chylous ascites is an uncommon finding in trauma. Although surgery may be indicated in selected patients, conservative treatment can be effective in most patients, with or without abdominal drainage. A high-protein and low-fat diet, with medium-chain triglycerides, is the indicated dietary regimen to decrease the amount of lymphatic fluid produced.

**Conclusion:**

Chylous ascites, although rare in trauma patients must be considered in the diferential diagnosis of free peritoneal fluid. Conservative treatment should be considered in the majority of cases reserving invasive treatments for specific situations.

## Highlights

• Chylous ascites is a rare condition, especially in abdominal trauma.
• A milky, odourless and turbid fluid is characteristic of chylous ascites.
• A high protein and low-fat diet is cornerstone in the management of these patients.

## 1 Introduction

Chylous ascites is the accumulation of a milk-like peritoneal fluid rich in triglycerides due to the presence of intestinal lymph in the abdominal cavity. It develops when there is a disruption of the lymphatic system due to traumatic injury or obstruction [1]. The lymphatic drainage from the intestinal lymph vessels along with the lumbar trunk drainage and the lymphatic drainage of the lower thorax drain into the cisterna chyli [2]. The cisterna chyli is located in the retroperitoneum, at the level of L1-2 vertebra and continues cephalically towards the venous system in the form of the thoracic duct which subsequently drains into the major venous system [2].

The most common causes of chylous acites in adults, in developed countries, are abdominal malignancy (one third are lymphomas) and cirrhosis, whereas in developing countries infectious diseases (tuberculosis and filariasis) predominate [2,3]. Other causes of chylous ascites are iatrogenic injury during surgical procedures (such as abdominal aortic aneurysm repair, retroperitoneal lymph node dissection, pancreaticoduodenectomy, liver transplantation and laparoscopic Nissen fundoplication) and blunt abdominal trauma [1].

The aim of this case report is to document a rare clinical presentation of chylous ascites due to blunt abdominal trauma. This case report has been elaborated in accordance with the SCARE criteria [4].

## 2 Clinical case

**Fig. 1:**
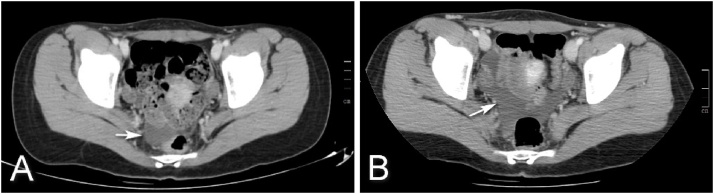
Computed tomography image on admission (image A) and after 24 h surveillance (image B) with an increase in the amount of peritoneal fluid (arrow).

A 27-year-old female and caucasian patient was admitted to the emergency department (ED) with abdominal pain due to a deceleration-type traffic accident (where she took the front passenger seat). The patient had no prior medical or surgical history and didn’t take any regular medication. In the objective examination abrasive lesions of the chest or lower quadrants of the abdomen were observed, caused by the seat belt, and she complained of a continues abdominal pain in the abdominal lower quadrants but had no signs of peritoneal irritation. In the initial approach of the patient in the ED, a hemogram and biochemical study, chest, vertebral column and pelvis x-ray, as well as an abdomino-perineal computed tomography (CT) scan were performed. During ED surveillance, although hemodynamically stable, the patient presented a progressive decrease in hemoglobin levels (13,3–10,4 mg/dL) in a 10 h interval and an increase in free intra-abdominal fluid detected on a second CT scan (Fig. 1). No other relevant analytical or imagiological findings were found, including fractures.

**Fig. 2:**
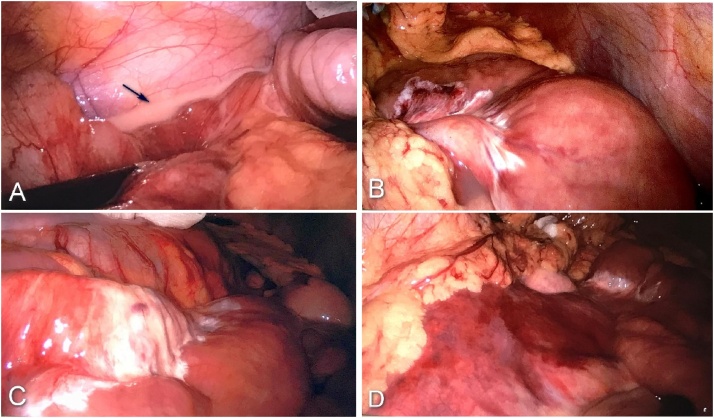
Exploratory laparoscopy image showing milk-like peritoneal fluid (image A – arrow) and mesenteric infiltration by the same fluid (image B and C). Image D shows a retroperitoneal hematoma.

After clinical case evaluation by the surgical team and management option discussion with the patient, informed consent was obtained for an exploratory laparoscopy by a general surgery assistant with experience in emergency surgery. The patient was placed in the supine position and a 12 mmHg pneumoperitoneum was created. Abdominal exploration was made using a 10 mm umbilical port (camera) and two 5 mm, right and left flank, ports. Exploratory laparoscopy revealed a milky-looking peritoneal fluid (Fig. 2), small stable and non-pulsatile retroperitoneal (left zone II and zone III) haematoma and a transverse colon deserosed segment. Intraoperatively, approximately 150 ml of milk-type peritoneal fluid was aspirated and the abdominal cavity was drained. The diagnosis of chylous ascites was confirmed by the determination of increased triglyceride levels (5 142 mg/dL) in the peritoneal fluid.

A low-fat diet with long-chain triglyceride restriction was started in the post-operative period and the patient presented a progressive daily decrease in abdominal drainage: 140 – 115 – 50 – 15 mL/24 h. The patient was discharged on the fifth post-operative day with a favorable clinical and analytical progression. There were no complications in the postoperative period. The patient was subsequently observed on a regular basis and after a one year follow-up period had no recurrence of chylous ascites.

## 3 Discussion

Chylous ascites is a rare condition, especially in abdominal trauma. It was first described by Morton in 1684 [5] and has an incidence of approximately 1 in 20,000 admissions at a large university-based hospital, over a 20-year period [6]. Studies show a mortality rate of chylous ascites in the range of 40–71%, increasing to 90% when of neoplasic origin [3].

The hyperextension and flexion movement of the trunk is the main cause of the lymphatic channel lesions in abdominal trauma which result in the accumulation of intra-abdominal or retroperitoneal lymph. Identification and ligation of injured lymphatic vessels is only possible in approximately 50% of these patients [5]. The isolated lesion of the lymphatic system without injuries to other organs is rare and to our knowledge only 10 clinical cases of such traumatic injuries with isolated chylous ascites have been described in the literature to date [1,5].

Chylous ascites is often asymptomatic with progressive abdominal distension and later abdominal discomfort which set in over weeks or months depending on the underlying cause [1,3]. In traumatic or post-operative cases there is a faster progression of the clinical scenario.

Ascitic fluid paracentesis is the main diagnostic test for the characterization of the peritoneal fluid [1]. Ascitic fluid should be sent for cell count and culture, Gram staining, total protein concentration, albumin, glucose, lactate dehydrogenase, amylase and triglyceride level determination and cytology analysis. The milky, odourless and turbid appearance of the peritoneal fluid is characteristic of chyle, and a level of triglycerides above 200 mg/dL is critical for the diagnosis of chylous ascites [[1], [2], [3]]. CT is important for the differential diagnosis of the etiology of chylous ascites, allowing identification of pathologic intra-abdominal masses or lymph nodes, as well as the extension and location of the collections [3]. In CT scans the ascitic liquid has a density similar to that of water and when the patient remains standing for a long period of time a hydro-fatty level is formed which is pathognomonic for this pathology [1,2]. Lymphoscintigraphy and lymphangiography are invasive tests that allow the identification of lymphatic leaks or fistulas and may be required when other tests fail to show the injured site [1,3].

Although the hematic density of hemoperitoneum in CT is different from that of lymph, in the present case of blunt abdominal trauma associated with a drop in haemoglobin levels and an increase in peritoneal fluid on CT scan lead to the surgical team decision and patient informed consent of an exploratory laparoscopy. The unmistakable visual characteristics of the peritoneal fluid associated with the increase in triglyceride levels in the fluid (5 142 mg/dL) confirmed the diagnosis of chylous ascites.

Conservative management with a high protein and low-fat diet, with medium chain triglycerides, should be started as soon as the diagnosis is made, with the aim of decreasing intestinal lymph production. The lymph transports lipids in the form of chylomicrons which originate from the breakdown of long chain triglycerides (converted into monoglycerides and free fatty acids, which become soluble in the form of chylomicrons). The medium and short chain triglycerides are in turn absorbed by the portal venous system, without involvement of the lymphatic system. Thus, decreased intake of long-chain triglycerides results in decreased production of intestinal lymph and consequently in the resolution of the lymphatic fistula. Studies give preference to enteral nutrition with a restricted diet of medium chain triglycerides, over total parenteral nutrition, which can be implemented when the patient does not respond to enteral nutrition [7].

Somatostatin and its analogue octreotide (100–200 μg 3 times daily, subcutaneous) can be associated to conservative treatment for their mechanism of action: they decrease intestinal absorption of fat, lower triglyceride concentration in the thoracic duct, and attenuate lymph flow in the major lymphatic channels [1,3,8]. Pan et al. demonstrated that the addition of somatostatin to conservative treatment was associated with a reduction in the length of hospital stay, the time for abdominal drain removal and the time to resume an oral diet [7].

When the patient doesn’t respond to conservative treatment in 2 weeks or maintains drainage volumes of more than 1000 mL per day for more than five days, invasive treatment, percutaneous or surgical, may be considered [5]. Loss of chyle in the peritoneal cavity results in the loss of essential proteins, lipids, immunoglobulins, vitamins, electrolytes and water, so it is important to manage metabolic or nutritional complications early and to consider invasive treatments in the absence of clinical improvement with conservative management [2]. Source identification of a lymphatic fistula may be very difficult so it is imperative that a pre-procedural image, with a lymphoscintigraphy or lymphangiography, be done in order to identify the lesion before invasive treatment.

We present a case report of a rare presentation of chylous ascites in a trauma patient. The patient underwent an exploratory laparoscopy due to the possibility of a hemoperitoneum associated to blunt abdominal trauma. The surgical intervention consisted of abdominal exploration, peritoneal fluid aspiration and abdominal drainage. No intervention was performed on the source of the chylous ascites. A high protein and low-fat diet, with medium chain triglycerides, was administered in the postoperative period which allowed lymphatic fistula resolution. Chylous ascites should therefore be considered in the differential diagnosis of free peritoneal fluid in trauma victims even in the absence of other lesions.

## 4 Conclusion

Chylous ascites is an uncommon finding in trauma with few cases published in the literature. Although invasive management, percutaneous or surgical, may be indicated in selected patients, conservative treatment is effective in a significant number of patients. A high-protein and low-fat diet, with medium-chain triglycerides, is the recommended dietary regimen to decrease the amount of lymphatic fluid produced.

## Declaration of Competing Interest

The authors report no declarations of interest.

## Funding

None funding for the research.

## Ethical approval

It does not need ethical approval in the institution.

## Consent

Written informed consent was obtained from the patient for publication of this case report and accompanying images.

## Author’s contribution

Vânia Fernandes did the conceptualization, data collection, surgical therapy for this patient, and writing the original draft.

Jacinta Queirós did surgical therapy for this patient and wrote the manuscript—review and editing.

Carlos Soares did surgical therapy for this patient and wrote the manuscript—review and editing.

## Registration of research studies

Not applicable.

## Guarantor

Vânia Fernandes.

## Provenance and peer review

Not commissioned, externally peer-reviewed.
